# Evidence for the efficacy of Tai Chi for treating rheumatoid arthritis: an overview of systematic reviews

**DOI:** 10.1590/1516-3180.2020.0346.R1.18112020

**Published:** 2021-03-03

**Authors:** Aline Mizusaki Imoto, Fábio Ferreira Amorim, Henderson Palma, Império Lombardi, Ana Lúcia Salomon, Maria Stella Peccin, Helbert Eustáquio Cardoso da Silva, Eduardo Signorini Bicas Franco, Leila Göttems, Levy Aniceto Santana

**Affiliations:** I PhD. Physiotherapist and Professor, Professional and Academic Master’s Program, Laboratory for Evidence-Based Healthcare, Escola Superior em Ciências da Saúde (ESCS), Brasília (DF), Brazil.; II PhD. Physician and Medical Education Manager, Undergraduate Medical Course, Escola Superior em Ciências da Saúde (ESCS), Brasília (DF), Brazil; Professor, Academic Master’s Program, Escola Superior em Ciências da Saúde (ESCS), Brasília (DF), Brazil; and Professor, Family Health Master’s Program (ProfSaúde), Escola Superior em Ciências da Saúde (ESCS), Brasília (DF), Brazil.; III MSc. Collaborative Researcher, Interdisciplinary Postgraduate Program on Health Sciences, Universidade Federal de São Paulo (UNIFESP), São Paulo (SP), Brazil.; IV PhD. Associate Professor, Interdisciplinary Postgraduate Program on Health Sciences, Department of Human Movement Sciences, Universidade Federal de São Paulo (UNIFESP), Santos (SP), Brazil.; V PhD. Nutritionist and Professor, Professional and Academic Master’s Program, Escola Superior em Ciências da Saúde, Brasília (DF), Brazil.; VI PhD. Physiotherapist. Associate Professor, Department of Human Movement Sciences, Universidade Federal de São Paulo (UNIFESP), Santos (SP), Brazil.; VII MSc. Dentist, Professional and Academic Master’s Program, Laboratory for Evidence-Based Healthcare, Escola Superior em Ciências da Saúde, Brasília (DF), Brazil.; VIII MSc. Physiotherapist and Doctoral Student, Department of Evidence-Based Healthcare, Universidade Federal de São Paulo (UNIFESP), São Paulo (SP), Brazil.; IX PhD. Professor, Professional and Academic Master’s Program, Laboratory for Evidence-Based Healthcare, Escola Superior em Ciências da Saúde (ESCS), Brasília (DF), Brazil.; X PhD. Coordinator, Professional Master’s Program, Laboratory for Evidence-Based Healthcare, Escola Superior em Ciências da Saúde (ESCS), Brasília (DF), Brazil.

**Keywords:** Tai Ji, Arthritis, rheumatoid, Exercise therapy, Mind-body exercise, Tai Chi Chuan, Rheumatoid arthritis exercise

## Abstract

**BACKGROUND:**

Rheumatoid arthritis (RA) is a chronic disease with higher prevalence among women aged between 30 and 50 years and general prevalence of 1% worldwide. Interventions promoting improvement of quality of life for individuals with RA are required. Tai Chi appears to be a low-cost alternative, with studies showing positive results from this technique. However, regarding aspects of RA such as pain and sensitivity, studies remain inconclusive.

**OBJECTIVES:**

To compare the effectiveness of the Tai Chi method for treating patients diagnosed with rheumatoid arthritis, among systematic reviews.

**DESIGN AND SETTING:**

Overview of systematic reviews with Cochrane and non-Cochrane methodology.

**METHODS:**

Systematic reviews involving quasi-randomized and randomized clinical trials (RCTs) on use of Tai Chi, with no restrictions regarding the date and language of publication, were included.

**RESULTS:**

Three systematic reviews were included. The effects of Tai Chi associated with education and stretching exercises versus education and stretching were evaluated in these reviews. They showed that improvements in the variables of mood, depression and functional index were associated with use of Tai Chi.

**CONCLUSIONS:**

The findings suggest that clinical improvement was achieved, although not statistically significant with regard to pain and disease pattern, as assessed using the ACR20 measurement. Improvements relating to disability and quality of life were also seen. There was a low level of evidence and therefore caution in data analysis is recommended. The three studies included showed poor reliability for providing an accurate and complete summary of use of Tai Chi among people diagnosed with rheumatoid arthritis.

**PROSPERO:**

CRD42019125501.

## INTRODUCTION

Rheumatoid arthritis (RA) is a chronic inflammatory systemic disease that mainly affects the musculoskeletal system. Its prevalence worldwide is 1%.^1^^,^^2^ Its occurrence rate peaks at ages between 30 and 50 years and it is primarily seen in females and people with a family history of the disease. Fifty percent of the risk of developing rheumatoid arthritis is attributed to genetic factors.^1^^,^^3^ Among environmental factors, the biggest trigger is smoking.^3^

Rheumatoid arthritis is further defined as an autoimmune condition, due to expression of autoantibodies such as the rheumatoid factor, which attacks a particular part of immunoglobulin G.^4^ Consequently, an inflammatory process occurs, thereby leading to proliferation of synovial cells in joints. The proliferating synovial inflammatory tissue is called “pannus” and leads to destruction of the adjacent cartilage and to bone erosion. Large production of pro-inflammatory cytokines, including tumor necrosis factor and interleukin-6, drives the destruction process,^1^ which is associated with persistent pain, deformities and disability, which leads to functional decline and generates social costs.^5^^,^^6^

There is a direct association between disease severity and higher treatment costs. It has been estimated that in the United States, US$ 2,000 to US$ 10,000 is spent per patient per year. In addition to this, there are indirect costs that are expected to be at least similar to these figures.^7^ De Azevedo et al.^8^ estimated the indirect costs of rheumatoid arthritis in a survey conducted at the Federal University of São Paulo and found that the costs ranged from US$ 466,107.81 to US$ 2,423.51 per patient per year. Based on this scenario, interventions that help to mitigate this disease form relevant strategies, both for patients and for public health.^9^

Positive effects on rheumatoid arthritis management through physical exercise programs aimed at maintaining muscle strength, mobility, flexibility, balance, resistance, and aerobic capacity have been demonstrated.^10^^,^^11^^,^^12^^,^^13^ Such exercises are generally prescribed at low intensities and are adapted to the demands of each patient.^14^ In this context, activities that involve both body and mind have shown positive results, as is the case of Tai Chi.

Tai Chi is a Chinese martial art composed of slow and smooth movements that reproduce shapes and postures inspired by nature, with circular and rhythmic movements and great mental focus.^14^^,^^15^ The intensity of Tai Chi practice is equivalent to walking at a speed of six kilometers/hour, and this gives rise to a moderate increase in heart rate.^2^ Practicing Tai Chi improves balance and postural control, increases lower limb strength, improves flexibility and prevents falls, especially among the elderly, in addition to promoting interaction between body and mind.^16^^,^^17^^,^^18^ Biopsychosocial benefits have also been shown, with improved wellbeing and reduced stress, anxiety, depression and mood disorders.^19^ In addition to the points mentioned above, because Tai Chi is a form of exercise that involves unloading of bodyweight, it has the benefit of stimulating bone formation, thus decreasing the risk of osteopenia and osteoporosis.^20^

Although studies have shown positive results from use of Tai Chi, the evidence regarding its effectiveness in treating rheumatoid arthritis remains limited and inconclusive with regard to aspects such as pain, function, sensitivity and edema. Hence, further studies to analyze the effects of this technique are required.

## OBJECTIVE

The purpose of this overview was to compare the effectiveness of the Tai Chi method among patients diagnosed with rheumatoid arthritis, among systematic reviews (SRs).

## METHODS

### Design

This overview included systematic reviews that used either Cochrane or non-Cochrane methodology, involving randomized clinical trials (RCTs) and quasi-randomized trials. There were no restrictions regarding the date or language of publication.

### Inclusion criteria

#### Types of participants

Only systematic reviews on patients diagnosed with rheumatoid arthritis in accordance with the American College of Rheumatology (ACR) criteria, with the diagnostic confirmation clarified in the body of the text, were included. There were no age or sex restrictions, regardless of the time of the disease onset,

#### Types of interventions

Systematic reviews that included the Tai Chi technique as a form of intervention, whether for prevention or treatment, in comparison with other conservative methods or placebo or no treatment, were assessed.

#### Types of outcomes

All outcomes involving Tai Chi practice among patients diagnosed with rheumatoid arthritis that were reported in the studies included were considered.

#### Process of searching for and selecting studies

The searches were conducted in September 2019, using the official terminology of the Health Sciences Descriptors (DeCS) and Medical Subject Headings (MeSH) databases. The search strategy is presented in Table 1. The following databases were accessed: Medline via PubMed, Cochrane Library, EMBASE and Virtual Health Library (VHL). The grey literature was also accessed. A manual search on the reference lists found in studies previously included was performed. Two independent reviewers (EF and HP) selected the studies, while observing the inclusion criteria mentioned above. Software available through the Rayyan website^21^ was used to remove duplicates and to make the final selection of studies. In cases of disagreement between the reviewers regarding the inclusion of specific studies, a third reviewer (AI) was included for making a final decision.

**Table 1.** Search strategy

Database	Search strategy
Virtual Health Library	(tw:(“Artrite Reumatoide” OR mh:c05.550.114.154* OR mh:c05.799.114* OR mh:c17.300.775.099* OR mh:c20.111.199* )) AND (tw:(“Tai Ji” OR (t’ai chi) OR (tai chi) OR (tai chi chuan) OR (tai-ji) OR (taiji) OR (taijiquan) OR mh:e02.190.525.890* OR mh:e02.779.474.913* OR mh:i03.450.642.845.560.500* )) AND (db:(“MEDLINE” OR “IBECS”))
PubMed	(((“Arthritis, Rheumatoid”[Mesh] or Rheumatoid Arthritis))) AND ((“Tai Ji”[Mesh] or Tai-ji or Tai Chi or Chi, Tai or Tai Ji Quan or Ji Quan, Tai or Quan, Tai Ji or Taiji or Taijiquan or T’ai Chi or Tai Chi Chuan))
Cochrane	#1 MeSH descriptor: [Arthritis, Rheumatoid] explode all trees
	#2 Rheumatoid Arthritis
	#3 #1 or #2
	#4 MeSH descriptor: [Tai Ji] explode all trees
	#5 Tai-ji or Tai Chi or Chi, Tai or Tai Ji Quan or Ji Quan, Tai or Quan, Tai Ji or Taiji or Taijiquan or T’ai Chi or Tai Chi Chuan
	#6 #4 or #5
	#7 #3 AND #6
EMBASE	‘rheumatoid arthritis’/exp AND ‘tai chi’/exp AND [embase]/lim

Data extraction was performed by two independent reviewers (HP and EF), through accessing the full published texts. The authors were contacted directly if the full text was not available. Data compilation was performed using the Review Manager 5.3 (RevMan) software (Copenhagen: The Nordic Cochrane Centre, Cochrane Collaboration, 2014).

The risks of bias and quality of evidence assessed per outcome were extracted from the analyses that had been made in the original systematic reviews, when available. Methodological quality was assessed by two independent reviewers (SP and EF) using the tool “Assessing the Methodological Quality of Systematic Reviews 2” (AMSTAR 2). Quantitative analyses using continuous variables were grouped as those expressed as a mean difference (MD) or as those expressed as a standardized mean difference (SMD), with 95% confidence intervals (CI). Analyses involving dichotomous outcomes were grouped according to the relative risk (RR) with the respective 95% confidence interval, when available in the original review. The I² value was calculated and was found to present heterogeneity.

## RESULTS

**Figure 1.**
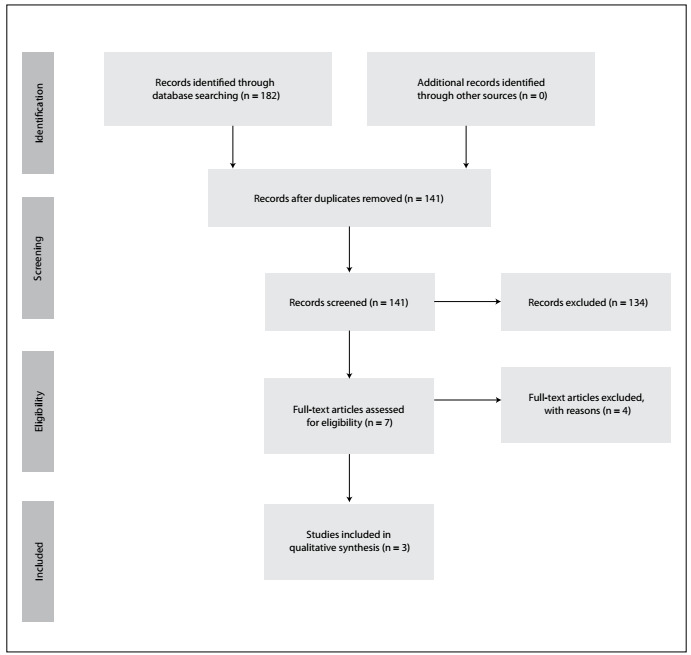
Flow diagram of literature searched and selection criteria.

The search strategy found 182 studies **(**Figure 1**)**, out of which 134 were excluded because they did not meet the inclusion criteria. Initially, seven studies were included for qualitative synthesis. However, full-text analysis showed that three of them were incompatible with the interventions mentioned in their abstracts and these were therefore excluded from the final analysis. Among the remaining studies, it was not possible to obtain the full text of one of them. An e-mail was sent to the authors, to request this article, but without any response. Therefore, we were left with three studies for the final analysis.^22^^,^^23^^,^^24^

### These systematic reviews were divided into different groups according to the intervention that was used.

Tai Chi in association with education and stretching exercises, versus education and stretching alone

The systematic review by Lee et al.^24^ included two randomized clinical trials that investigated the effectiveness of the Tai Chi technique among people diagnosed with rheumatoid arthritis. In one of these randomized clinical trials, Tai Chi was applied in association with education for the patients combined with stretching exercises. The outcomes of pain, disability index and quality of life were assessed. The control group was formed by 10 volunteers who received educational instructions relating to the symptoms of rheumatoid arthritis and nutrition focused on this disease (40 minutes) plus stretching exercises (20 minutes), twice a week for 12 weeks. The intervention group participated in Tai Chi classes lasting 60 minutes, twice a week for 12 weeks. The group that practiced Tai Chi presented improvements in their disability index (P = 0.01) and quality of life (P = 0.01). However, no significant difference regarding pain was observed.

Regarding the pain outcome, neither of the studies showed any significant change in comparison with the control.^25^^,^^26^ In one randomized clinical trial, there was a significant difference between the groups regarding the depression and mood assessments,^25^ in comparison with the control, while in the other randomized clinical trial there was an improvement in the assessment through the profile of the mood state inventory.^26^ This latter randomized clinical trial showed improvement in the intervention group regarding the functional index.^26^ In relation to quality of life, the assessment in this second randomized clinical trial showed that Tai Chi was favorable for the aspect of vitality, compared with the control.^26^

In the systematic review by Macfarlane et al.,^22^ the effectiveness of several complementary and alternative therapies for treating rheumatoid arthritis, including Tai Chi, was ascertained. One randomized clinical trial was included in their review, which was the same study as above, in which Tai Chi exercises were applied for 60 minutes, twice a week for 12 weeks. The control group was composed of 20 people who received guidance on nutrition and information regarding the disease for 40 minutes plus stretching exercises for 20 minutes, twice a week. Among the 13 outcomes assessed, 10 did not show any statistically significant change between the groups, including pain, change in the overall assessment, joint swelling, sore spots, fatigue and functional capacity. There were considerable improvements in vitality, mood and skills in the intervention group. No significant differences in laboratory tests such as erythrocyte sedimentation rate (ESR) and C-reactive protein (CRP) were found.

### Tai Chi versus control (not practicing Tai Chi)

In the systematic review published by Mudano et al.,^20^ the effect of Tai Chi for treating rheumatoid arthritis treatment was analyzed. Out of all the studies assessed in this review, seven studies with 345 participants were selected, comprising 180 individuals who received Tai Chi intervention and 165, other treatments. The following outcomes were analyzed: pain, disease activity using a disease activity score, function, joint sensitivity, swelling, range of motion, handgrip strength, 50-foot walking test and ACR20. The ACR20 measurement of clinical improvement is defined as a 20% improvement in three out of the following five criteria: patient overall assessment; physician overall assessment; functional ability measurement, visual analogue pain scale (VAS); and erythrocyte sedimentation or C-reactive protein rate.

Based on the studies used in this review, which were of low quality, the authors indicated that was not possible to affirm that use of Tai Chi resulted in an improvement in pain, as measured using a visual analogue pain scale. This was despite a mean difference (MD) in VAS score of −2.15 (95% CI −3.19 to −1.11) through its use, which may have been clinically relevant. The results regarding disease activity and functional ability, measured using the Health Assessment Questionnaire (HAQ) (MD −0.33; 95% CI −0.79 to 0.12), were also inconclusive. Regarding the disease pattern assessed using ACR20, there was no statistically significant result, but the difference may have been clinically relevant. Thus, the result for this outcome was also inconclusive: Tai Chi group (RR = 11.0; 95% CI 0.69 to 175.86); with 50% absolute difference (95% CI 18% to 82%). Likewise, the results regarding the outcomes of sensitivity, swelling, range of motion, handgrip strength and walking test were inconclusive. The intervention program duration ranged from 8 to 12 weeks.

### Methodological quality assessment

The methodological quality assessment showed that among the three systematic reviews (Lee et al.,^24^ Macfarlane et al.^22^ and Mudano et al.^20^), only one (Mudano et al.^20^) presented high methodological quality (Table 2). According to AMSTAR 2, a systematic review has high quality in a situation of absence or presence of only one non-critical item. Thus, AMSTAR 2 provided an accurate understanding of the results from the studies included.

**Table 2.** AMSTAR 2 assessment of the studies included

Questions AMSTAR 2	1	2	3	4	5	6	7	8	9	10	11	12	13	14	15	16
Mudano et al.^20^	Yes	Yes	Yes	Yes	Yes	Yes	Yes	Yes	Yes	No	Yes	No	Yes	Yes	Yes	Yes
Lee et al.^23^	Yes	No	Yes	No	No	Yes	No	No	No	No	*	*	Yes	No	*	Yes
Macfarlane et al.^21^	Yes	No	Yes	Yes	Yes	No	No	Yes	No	No	*	*	No	No	*	Yes

*No meta-analysis conducted

The other two systematic reviews were considered to be of low methodological quality. According to Shea et al.,^27^ studies of low quality do not present sufficient reliability to provide an accurate and complete summary of the data. AMSTAR 2 defines the following as critical domains: protocol registered before commencement of the review; adequacy of the literature search; justification for excluding individual studies; risk of bias from the individual studies included; appropriateness of meta-analysis methods; consideration of risk of bias in interpreting the results from the review; and assessment of the presence and likely impact of publication bias. The studies included are described in Table 3.

**Table 3.** Description of the studies included

Study ID	Objective of the study	Number of articles included	Outcomes and results
Mudano et al.^20^	To assess the benefits and harm of Tai Chi as a treatment for people with rheumatoid arthritis.	Seven RCTs with 345 participants	The majority of the trials presented high risk of performance bias and detection bias, due to the lack of blinding of participants or assessors. The duration of the Tai Chi programs ranged from 8 to 12 weeks. It was uncertain whether Tai Chi-based exercise programs provided any clinically important improvement in pain among Tai Chi participants, compared with no therapy or alternate therapy. There was very low-quality evidence, which was downgraded in relation to blinding and attrition. The evidence was inconclusive with regard to any significant difference in disease activity. Regarding assessment of function, the change in mean score in the Health Assessment Questionnaire for the Tai Chi group was an 11% absolute improvement, but with very low-quality evidence. The authors were unsure whether there had been any significant improvement, given that the results were inconclusive.
Lee et al.^23^	To update and evaluate the clinical trial evidence for the effectiveness of Tai Chi for patients with rheumatoid arthritis.	Five studies: 2 RCTs and 3 CCTs	Pain: Two RCTs suggested that there was no significant reduction in pain, compared with education plus stretching exercise and usual activity. One CCT suggested that there was significant pain reduction, compared with the usual activity control group. Fatigue: One RCT showed that there was no improvement, compared with usual activity. One CCT compared use of Tai Chi with usual activity and suggested that use of Tai Chi was effective in relation to fatigue. Range of motion and joint functions: Two CCTs did not show any intergroup differences with regard to joint tenderness and the number of swollen joints, compared with usual activity. Depression and mood: One RCT reported that there was a significant intergroup difference in depression, compared with education plus stretching exercise. One RCT compared use of Tai Chi with usual activity and suggested that its use led to improvement of mood in the profile of mood state inventory. Functional index: One RCT compared use of Tai Chi with education plus stretching exercise and reported that its use was favorable regarding the disability index. One CCT reported that there was no improvement regarding the ability to perform activities of daily life. Quality of life: One RCT tested the effectiveness of Tai Chi regarding quality of life and reported that its use led to an intergroup difference on the vitality subscale of the SF36 questionnaire, compared with education plus stretching exercise.
Macfarlane et al.^21^	To review the evidence from RCTs relating to management of rheumatoid arthritis with complementary therapy ( not taken orally or applied topically).	11 RCTs included, but only one study about Tai Chi	Use of Tai Chi led to significantly greater improvement in terms of disability, vitality and mood. There were no significant differences between the groups regarding 10 of the 13 outcomes measured, including pain (past week and current), patient’s overall assessment of change, swollen joints, tender points, fatigue and functional capacity.

RCT = randomized clinical trial; CCT = controlled clinical trial.

## DISCUSSION

The purpose of this overview was to ascertain the effectiveness of the Tai Chi method used among people who had been diagnosed with rheumatoid arthritis. Through the systematic search and application of the inclusion and exclusion criteria, three systematic reviews on the use of the Tai Chi method for treating rheumatoid arthritis were included.

Lee et al.^24^ showed that use of Tai Chi was beneficial regarding the outcomes of disability and quality of life. However, there was no difference in the pain outcome. In the study by Mudano et al.,^20^ which was a Cochrane systematic review, use of the Tai Chi method improved the parameters of pain and disease pattern, as assessed using ACR20. There was no statistical difference; however, because the outcome levels were lower, the difference may have been clinically relevant. Other results were also considered inconclusive, such as sensitivity, swelling, range of motion, handgrip strength and walking test. The review authors reported that the articles were of poor quality. Thus, the effects of the Tai Chi method with regard to improvement of rheumatoid arthritis patients’ condition remain inconclusive.

Slight increases in parameters such as pain and disease activity may have been due to the condition of the patients included in the study. Given that Tai Chi requires balanced and controlled movements, patients who can practice this type of exercise often do not present pain as the main symptom.^28^ This may have been the reason why there was no statistically significant difference in the pain outcome after Tai Chi programs. The same explanation can be put forward in relation to inconclusive outcomes such as the number of painful and edematous joints.

Regarding improvements in disability and quality of life, authors like Wang^28^ have reported that Tai Chi is associated with reduced stress, anxiety and depression, as well as improved quality of life.

The methodological quality assessment demonstrated that the review by Mudano et al.,^20^ published by the Cochrane Collaboration, was superior regarding most of the AMSTAR 2 methodological aspects. It is important to highlight critical points that were not addressed by Lee et al.^24^ and Macfarlane et al.,^22^ in the other systematic reviews included, such as the lack of protocol registration before the commencement of the review and the lack of risk-of-bias assessment regarding the individual studies included. Following the previously published protocol reduces the risk of bias, while a risk-of-bias assessment is extremely relevant, because bias may be present in the design, planning, conduction and analysis of clinical trials.

In the studies included, practicing Tai Chi led to positive results regarding improvement of disability, quality of life, depression, mood and vitality. Regarding pain, one of the main symptoms of rheumatoid arthritis, these studies did not show any clear benefits.

Based on this overview, in analyzing the use of Tai Chi for treating conditions such as dementia in a population that was considered to be of senior age, its use gave rise to improvement in cognitive functions, visuospatial skills, semantic memory and verbal learning, thus leading to improvement of mood, quality of life and, consequently, vitality.

Limitations such as difficulty in standardizing the methods used in Tai Chi practice and the diagnostic model for primary study samples made it impossible to include some studies in the qualitative result analysis of this overview.

The low number of systematic reviews included in the present overview and the low methodological quality of two out of these three systematic reviews further exemplify the limitations found. Based on the findings from the present overview, healthcare professionals should consider using this overview to improve possible symptoms in people diagnosed with rheumatoid arthritis, either in a rehabilitative or in a preventive manner, according to whether any symptoms have yet been exhibited.

The implication from the present overview is that studies with longer follow-up periods (more than six months) should be conducted. Furthermore, qualitative studies should be conducted to assess other aspects of the effect of Tai Chi on disorders such as anxiety, depression and stress.

## CONCLUSION

The present review identified three studies regarding use of Tai Chi among patients who had been diagnosed with rheumatoid arthritis. The findings suggest that its use led to clinical improvement, though not statistically significant regarding pain and disease pattern, as assessed using the ACR20 measurement. Moreover, there were improvements relating to disability and quality of life. Other outcomes, such as sensitivity, swelling, range of motion, handgrip strength and walking test, were inconclusive. Considering that among the three studies included only one presented high methodological quality, while the other two were of low quality, caution is needed in evaluating these data. The three studies included present poor reliability for providing an accurate and complete summary of use of the practice of Tai Chi among people diagnosed with rheumatoid arthritis.
